# Substantial differences in soil viral community composition within and among four Northern California habitats

**DOI:** 10.1038/s43705-022-00171-y

**Published:** 2022-10-13

**Authors:** Devyn M. Durham, Ella T. Sieradzki, Anneliek M. ter Horst, Christian Santos-Medellín, C. Winston A. Bess, Sara E. Geonczy, Joanne B. Emerson

**Affiliations:** Department of Plant Pathology, University of California Davis, Davis, CA USA

**Keywords:** Soil microbiology, Microbial ecology

## Abstract

Viruses contribute to food web dynamics and nutrient cycles in diverse ecosystems, yet the biogeographical patterns that underlie these viral dynamics are poorly understood, particularly in soil. Here, we identified trends in soil viral community composition in relation to habitat, moisture content, and physical distance. We generated 30 soil viromes from four distinct habitats (wetlands, grasslands, woodlands, and chaparral) by selectively capturing virus-sized particles prior to DNA extraction, and we recovered 3432 unique viral ‘species’ (dsDNA vOTUs). Viral communities differed significantly by soil moisture content, with viral richness generally higher in wet compared to dry soil habitats. However, vOTUs were rarely shared between viromes, including replicates <10 m apart, suggesting that soil viruses may not disperse well and that future soil viral community sampling strategies may need to account for extreme community differences over small spatial scales. Of the 19% of vOTUs detected in more than one virome, 93% were from the same habitat and site, suggesting greater viral community similarity in closer proximity and under similar environmental conditions. Within-habitat differences indicate that extensive sampling would be required for rigorous cross-habitat comparisons, and results highlight emerging paradigms of high viral activity in wet soils and soil viral community spatial heterogeneity.

## Introduction

Viruses are abundant across Earth’s ecosystems, contributing to microbial dynamics and biogeochemical cycles, yet they remain understudied, particularly in terrestrial habitats [1, 2]. Soil viral abundance measurements vary substantially, ranging from nearly zero in dry deserts to over 10^9^ virus-like particles per gram in wetlands [3]. In the better studied surface oceans, viruses lyse approximately 20% of microbial biomass daily, impacting nutrient and energy cycles [4], and recent work suggests that viruses may be similarly important in terrestrial ecosystems [3, 5–12]. For example, viruses have been suggested to affect carbon cycling in thawing permafrost peatlands by preying on methanogens and methanotrophs and by encoding glycoside hydrolases to break down complex carbon into simple sugars [2]. Soil viruses have been demonstrated to actively infect their hosts in a variety of soil environments, even in extreme environments such as arid deserts [5, 13], and soil viral communities can be spatially structured [14–16]. Despite these emerging ecological patterns, comparisons of soil viral diversity within and across habitats are limited.

Here, we compared dsDNA viromes (<0.2 µm viral size-fraction metagenomes representing the dsDNA viral community, presumably dominated by viruses of bacteria and archaea) [14] from four distinct habitats (wetlands, grasslands, chaparral shrublands, and oak woodlands) across five UC Davis Natural Reserves field sites within Northern California. We compared viral species (vOTU) richness, vOTU detection patterns, and viral community beta-diversity, according to habitat type, soil properties, and spatial distribution to better understand the fundamental relationships between soil viruses and the ecosystems that they inhabit.

## Results and discussion

To compare soil viral community composition within and across terrestrial habitats on a regional scale, viromes were generated from 34 near-surface (top 15 cm) soil samples, with a total of 30 viromes included in downstream ecological analyses (see Supplementary Methods). The analyzed viromes were collected from four distinct habitats (wetlands, grasslands, chaparral shrublands, and woodlands, each with 7, 14, 4, and 5 viromes, respectively) across five field sites (Fig. S1 for sampling scheme, Table S1 for soil properties). Following quality filtering, the 30 viromes generated an average of 72,950,833 reads and 416 contigs ≥10 Kbp per virome (Table S2). Wetland viromes yielded more contigs ≥10 Kbp than viromes from other habitats, both in total and on average per virome (Table S2). We used VIBRANT to identify 3490 viral contigs in our assemblies, which were clustered into 3,432 viral operational taxonomic units (vOTUs), defined as ≥10 Kbp viral contigs sharing ≥ 95% average nucleotide identity over 85% contig length [17]. Constrained analysis of principal coordinates (CAP analysis) revealed strong clustering by habitat rather than by site, implying that, where environmental parameters are substantially different, environmental conditions are stronger drivers of viral community composition than geographic distance (Fig. S2).

**Fig. 1:**
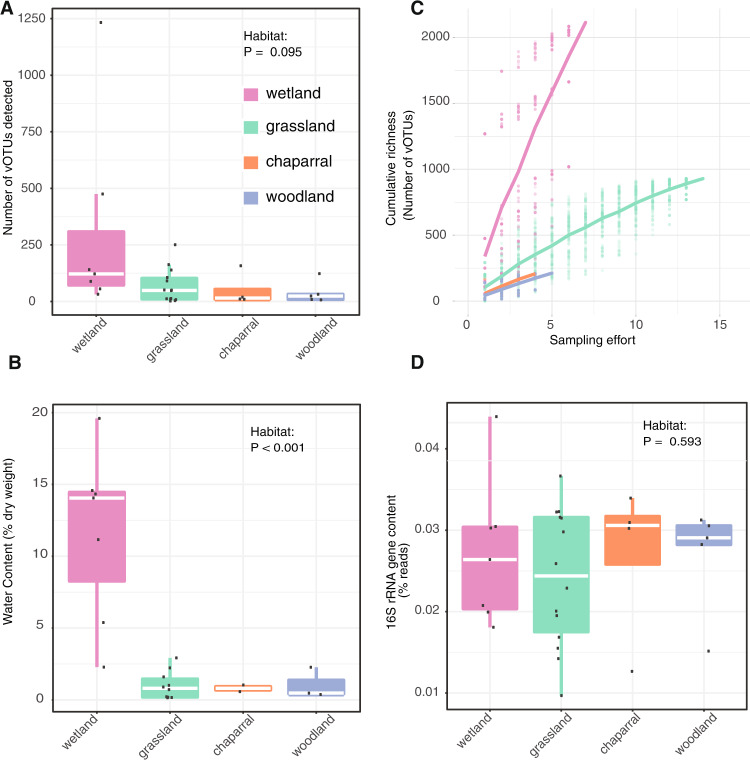
Evidence for higher viral richness in wetter soils. Comparisons between habitats of (**A**) viral richness (number of identified vOTUs with coverage along at least 75% of the contig in a given virome), dots represent richness in individual viromes, (**B**) accumulation curves of cumulative vOTU richness as sampling effort increased, dots represent cumulative richness at each sampling effort across 100 permutations of virome order; the overlaid lines display the mean cumulative richness per habitat, (**C**) water content, calculated as (wet weight—dry weight) divided by dry weight, and (**D**) bacterial 16S rRNA gene content in the viromes, based on percent of viromic reads mapping to 16S rRNA reference genes. VIBRANT [21] was used to identify 3,490 viral contigs in our assemblies, and these viral contigs were clustered at 95% average nucleotide identity (ANI) into 3432 viral operational taxonomic units (vOTUs). For (**A**), (**B**), (**D**), raw data are plotted on top of the box plots, with white lines showing the median, boxes indicating 75% of the data, whiskers extending to 90%, and points beyond the whiskers indicating outliers.

Multiple lines of evidence suggest that wetter soils harbored greater viral diversity than drier soils. We recovered the most vOTUs from wetlands, both in total (56% of all vOTUs were from wetlands) and per virome (on average, 307 vOTUs were recovered per wetland virome, compared to 116 from all habitats) (Fig. 1A). Unsurprisingly, wetlands had significantly greater moisture content than other habitats (Fig. 1B; ANOVA followed by Tukey multiple comparisons of means, *p* < 0.001), especially considering that soil samples were collected towards the end of the Mediterranean climate dry season, meaning that most habitats had not received precipitation for the preceding ~6 months. Although viral richness was highest in wetlands, this was not statistically significant (ANOVA model richness ~ habitat, *p* = 0.095). Still, nonparametric tests, which account for nonlinear correlations, revealed a significantly positive correlation between viral richness and soil moisture content (Spearman rho = 0.33, *p* = 0.036; Kendall tau = 0.22, *p* = 0.045). Viral community beta-diversity was also related to soil chemical properties overall (Mantel test, R^2^ = 0.43, *p* = 0.009; Table S1), while distance between sites only accounted for 5% of the variation (Partial Mantel test, R^2^ = 0.38, *p* = 0.009). Taken together, viral diversity was generally highest in wet soils.

We next wondered whether differences in sampling effort or bacterial content in the viromes could have produced the observed diversity patterns. For example, viral diversity could have appeared artificially higher in wetland habitats if wetland viral diversity was lower but well-sampled, compared to other habitats with higher diversity but poorer sampling. In a comparison of accumulation curves, such a pattern would include a more horizontal slope in the wetlands and a more steeply increasing vertical slope over sampling effort in the other habitats. We tested this by comparing accumulation curves across habitats, which revealed the opposite trend: wetlands exhibited the most steeply increasing slope and were thus likely to be the most under-sampled, in terms of true viral diversity (Fig. 1C). Given that relic DNA and small bacteria can pass through 0.22 µm filters, bacterial sequences are known to be present in viromes [18]. Although we had no specific reason to expect differences in the ratio of bacterial to viral DNA content among habitats, we wanted to exclude the possibility that viral diversity appeared artificially higher in wetlands due to a lower proportion of bacterial content in wetland viromes compared to other habitats. To compare bacterial content in viromes across habitats, 16S rRNA gene fragments were recovered from raw reads (Fig. 1D). The percentage of 16S rRNA gene sequences in each virome ranged from 0.01 to 0.044% (consistent with prior reports of 0.028% bacterial 16S rRNA gene content in similarly prepared viromes from agricultural soils [18]) and did not differ significantly by habitat (ANOVA, *P* = 0.595). Thus, viral diversity estimates did not seem to be disproportionately skewed by sampling effort or the presence of non-viral sequences in viromes. The higher diversity of viruses in wetlands compared to the other, drier habitats could be due to higher bacterial activity and therefore more susceptible hosts, leading to higher viral activity and diversity. Additionally, better soil connectivity in wet soil, leading to higher dispersion of free viruses and hosts, could increase the chances of virus-host encounters.

**Fig. 2:**
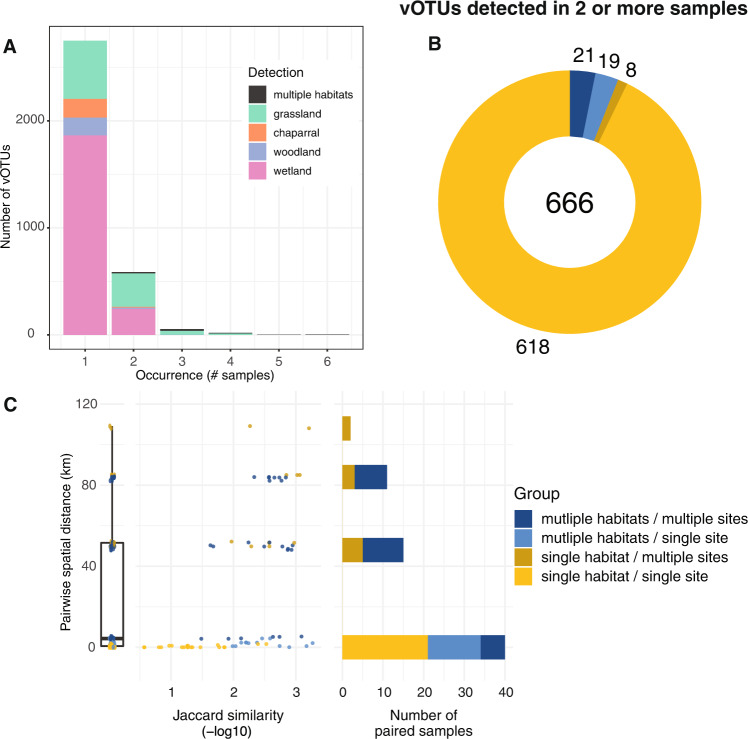
Substantial differences in vOTU detection and viral community composition between soil viromes, both within and across habitats. **A** vOTU detection patterns, in terms of the number of viromes (samples) in which each vOTU was detected (x-axis) and the number of vOTUs exhibiting a given detection pattern (y-axis), with stacked bar colors indicating the habitat(s) in which each vOTU was detected, (**B**) vOTUs detected in more than one virome, colored by their detection patterns across sites and habitats (legend to the right of panel C), numbers indicate total vOTUs (center) and vOTUs per detection pattern, and (**C**) Pairwise community compositional similarity (x-axis) by geographic distance (y-axis) between viromes, colored according to detection patterns across habitats and sites (legend on the far right). Left graph: box plot of pairwise distances for all viromes (condensed x-axis). Middle graph: Each point represents the -log10 Jaccard similarity between two viromes along the x-axis, with lower values (left side of the x-axis) indicating greater viral community similarity. Right graph: Total number of virome pairs included at each geographic distance. Paired viromes were only included if they shared at least one vOTU.

Perhaps the most striking result from this study was the uniqueness of each soil viral community. This feature has been previously highlighted in viruses identified in other environments, such as highly oligotrophic water [19] and hydraulic fracturing wells [8]. The majority of vOTUs (81%) in this regional study were only detected in a single virome (Fig. 2A, Fig. S3A). Of the 666 vOTUs detected in at least two viromes, 93% were detected in viromes from the same habitat and site (Fig. 2B, Fig. S3B). The most similar viral communities were from the same habitat and site (i.e., biological replicates) less than 1 Km apart (Fig. 2C). Within the same site, viral communities were less similar between habitats than within the same habitat. Additionally, viromes from the same habitat at different, nearby sites (within 6 Km) did not share any vOTUs, suggesting substantial differences in viral communities over local distances both within and between habitats. At greater distances, community similarity generally decreased, even between viromes from the same habitat (Fig. 2C). Still, 21 vOTUs were detected in multiple habitats across multiple sites (Fig. 2B), and some vOTUs were shared between the two farthest sites (109 Km apart, Fig. 2C), suggesting some degree of regional conservation of viral populations. Overall, results suggest substantial differences in soil viral community composition in the same habitat on the scale of meters, greater similarity of viral communities in close proximity and under similar environmental conditions, and a small number of vOTUs shared over regional distances.

Although soil viral communities are woefully under-sampled, making sweeping generalizations premature, results from this study and others converge on a picture of high local viral diversity, with communities differing substantially over space and by habitat, with little regional co-occurence of viral ‘species’ (vOTUs). Viral community distance-decay relationships have been observed across an 18-m long agricultural field [14] and in a 200 m^2^ grassland [20], consistent with the meters-scale differences in viral community composition between replicates from the same habitat observed in this study. However, the proportion of vOTUs shared over meters varied across these studies, with many vOTUs shared across the agricultural field but most vOTUs not shared between samples ~10 m apart in this study and in the grassland field, suggesting the potential for different spatial patterns in natural and managed soils, perhaps due to different amounts of soil mixing by management practices, such as tilling. Similarly substantial differences among viral communities on a regional scale were also identified in a study of grassland and peatland RNA viromes, which shared few viruses between samples [16]. However, ter Horst et al. showed that 4% of the vOTUs from a Minnesota, USA peatland were shared in other peatlands, often on different continents [15], consistent with the recovery of a small number of vOTUs shared over >100 Km distances here. Taken together, we propose that soil viral communities often display high heterogeneity within and among habitats, presumably due to a combination of host adaptations and microdiversity, dispersal limitation, and fluctuating environmental conditions over space and time.

## Supplementary information

Supplemental Methods

Supplementary Table S1

Supplementary Table S2

Supplementary Table S3

Supplementary Table S4

Supplementary Figures

## Data Availability

The datasets generated and/or analyzed in this study have been submitted to the NCBI sequence read archive (SRA) under BioProject number PRJNA831438 and will become available upon publication. The vOTU fasta sequences and R scripts are available on https://github.com/ellasiera/Nat_res_vOTUs.
