# Supplementary material for: Substantial differences in soil viral community composition within and among four Northern California habitats
Source: ISME Commun. 2022 Oct 13;2:100. doi: 10.1038/s43705-022-00171-y (PMC9723544; doi:10.1038/s43705-022-00171-y)
Supplement: Supplementary file 1 — Supplemental Methods [file 43705_2022_171_MOESM1_ESM.docx]

**Supplemental Methods**

*Experimental design adjustments on account of COVID-19 and wildfire*

The sample set analyzed in this study, collected in November 2019, was meant to be the first of several time points, with plans for periodic sampling of the same sites in 2020-2021. However, COVID-19 prevented sample collection throughout much of 2020, and then the LNU Complex Fires completely burned 2 and partially burned 1 of our 5 field sites in August 2020. Thus, the decision was made to analyze this November 2019 dataset on its own.

*Site description and field sampling*

Soil samples were collected from woodland, chaparral, grassland, coastal bluff, and wetland habits at five UC Davis Natural Reserves sites in Northern California, USA: Bodega Bay (BB), Jepson Prairie (JP), McLaughlin (ML), Quail Ridge (QR), and Stebbins Cold Canyon (SCC) (Sup. Fig 1). From November 14 through November 16, 2019, 34 samples were collected from 17 plots within the five sites. We sampled at least two distinct habitats from each site, and two samples (replicates) were retrieved from each plot, with each plot consisting of one contiguous habitat. Inter-sample distance between replicates in each plot ranged from 6.5-74.2 m and was meant to capture a large area within one instance of the habitat (*i.e.*, no distinct habitat types were permitted between replicate samples), considering available habitat space and accessibility. The number of samples per site and habitat, along with GPS coordinates (Supplementary Table S1) and distance between sites, appear in Supplementary Tables S3 and S4, and soil chemistry appears in Supplementary Table S1.

For each sample, three 7.6 cm diameter soil cores were collected from the 0-15 cm depth range (excluding aboveground biomass) with a slide-hammer corer, approximately 15 cm apart from each other. The three fresh cores for each sample were homogenized and transferred into sterile bags on site, kept on ice for 1-2 days in the field, and then passed through an 8 mm mesh sieve within 3 days of sample collection. 10 g of each sample were set aside for measuring soil moisture, and the remaining soil was stored at -80 °C prior to processing for DNA extraction and soil properties.

*Soil viral purification and DNA extraction*

Viral particle purification and DNA extraction proceeded similarly to the Santos-Medellin et al. [[1]](https://paperpile.com/c/hsgGpF/CBCq) protocol (which was largely derived from Goller et al. [2]), with slight modifications. Briefly, for each sample, a suspended soil solution was prepared by adding 9 mL of PPBS Buffer (2% bovine serum albumin, 10% phosphate-buffered saline, 1% potassium citrate, and 150 mM MgSO4) to 10 g of soil [[2]](https://paperpile.com/c/hsgGpF/s6I1). To elute virions, soil suspensions were shaken by inversion and placed in an orbital shaker for 20 min at 300 RPM and 4 °C, then centrifuged (Sorvall RC5C, Thermo-Fisher Scientific, Waltham, MA, USA) for 10 min at 10,000 x g and 4 °C. Supernatant was briefly stored at 4 °C while the soil pellet was resuspended with 9 mL of PPBS Buffer, and the entire process of mixing, shaking, and centrifugation was repeated two more times, resulting in a total of ~27 mL of supernatant per soil sample. To pellet and remove remaining soil, supernatant was centrifuged for 8 min at 5,000 x g and 4 °C, with supernatant transferred, excluding the pellet. This step was repeated twice. The pooled supernatants were filtered through a 0.22 µm polyether sulfone filter and centrifuged (Optima LE-80K ultracentrifuge, Beckman Coulter, Brea, CA, USA; 50.2 Ti rotor) for 2 hours 25 minutes at 32,000 x g and 4 °C. The supernatant was removed and the pellet containing viral particles was resuspended in 100 µL of ultrapure water. DNase was not used here, as samples were frozen, which may compromise the integrity of viral capsids [[3, 4]](https://paperpile.com/c/hsgGpF/7jx2+0eyE). However, ecological trends from viromes can still be detected without DNase treatment [[3, 4]](https://paperpile.com/c/hsgGpF/7jx2+0eyE).

For DNA extraction, 250 µL of phenol:chloroform:isoamyl alcohol (ratio 25:24:1, pH 8) was added to each 100 µL sample. The mixture was briefly vortexed and then incubated on ice for 3 cycles of 1 min on and 5 min off intervals. Samples were then centrifuged for 5 min at 14,000 x g at 4 °C and the supernatant was transferred to a Phase Lock Gel™ tube, and then centrifuged for 5 min at 14,000 x g at 4 °C. The viral DNA supernatant (100 µL) was mixed with 25 µL of 3 M sodium acetate, 1.5 µL of Glycoblue™ Coprecipitant, and 250 µL isopropanol. The mixture was vortexed briefly and then incubated at -80 °C for 20 min. After incubation, samples were centrifuged for 20 min at 14,000 x g at 4 °C. Supernatant was removed and the viral pellet was washed with 500 µL of 70% ethanol and centrifuged for 5 min at 14,000 x g at 4 °C. The ethanol was removed, and the samples were centrifuged for another minute, then left to dry for 5 min. The viral DNA pellet was resuspended with 100 µL of non-DEPC-treated ultrapure water. DNA yields were quantified using an Invitrogen Qubit 4 Fluorometer with 1X High Sensitivity DNA assay (Thermo Fisher Scientific, Inc., Waltham, MA, USA). Of the 34 samples from which viral DNA was extracted, one replicate from a BB wetland site (sample ID: BB_3_2) and one replicate from a QR woodland site (sample ID: QR_2_1) yielded insufficient DNA and were thus excluded, resulting in 32 samples remaining for library construction and sequencing.

*Library construction and sequencing*

Libraries from the 32 viromes were constructed with the KAPA Roche kit, as described in Santos-Medellin et al. [[1]](https://paperpile.com/c/hsgGpF/CBCq), pooled in equimolar concentrations, and sequenced to a target depth of 10 Gbp per virome on the Illumina NovaSeq platform at the UC Davis DNA Technologies Core.

*Bioinformatics*

Raw sequencing reads were quality-filtered and trimmed using Trimmomatic-0.39, as in Roux et al. [[5, 6]](https://paperpile.com/c/hsgGpF/gkZV+PMYh). Removal of PhiX147 sequences was performed with BBDUK from the BBMap package (version: BBMap-38.87) [[7]](https://paperpile.com/c/hsgGpF/OtJt), followed by assembly with MEGAHIT-1.2.9 in meta-large mode [[8, 9]](https://paperpile.com/c/hsgGpF/a23t+0hJh). Each sample was assembled separately, and contigs shorter than 10kb were removed. Viral contigs were predicted using VIBRANT-1.2.1 with default settings [[10]](https://paperpile.com/c/hsgGpF/15oU). dRep version 2.0.0 [[11]](https://paperpile.com/c/hsgGpF/etkR) was used for clustering sequences at 95% average nucleotide identity across 85% of the length of the shorter contig, resulting in a reference set of 3,432 viral population sequences (vOTUs). Reads from the 32 viromes were mapped to these reference vOTU sequences with Bowtie 2-2.4.2 [[12]](https://paperpile.com/c/hsgGpF/r3L4), using sensitive mode, and the vOTU read-mapped coverage table was created with BamM-1.7.3 [[13]](https://paperpile.com/c/hsgGpF/Xi2g). Where vOTU breadth (contig length fraction with at least 1x coverage depth) was below 75%, coverage was set to zero [[8]](https://paperpile.com/c/hsgGpF/a23t). Two viromes (sample IDs: BB_6_1 and BB_6_2) were excluded from downstream comparative analyses at this point because they were the only samples from a coastal bluff habitat across the dataset, thus this habitat was deemed insufficiently sampled for cross-habitat comparisons. The final dataset for comparative analyses consisted of 30 viromes.

16S rRNA gene abundance recovery from viromes was performed by identifying reads that map to this gene with SortMeRNA v4.2.0 [[15]](https://paperpile.com/c/MnOQcM/s2Ub) against the SILVA database [[16]](https://paperpile.com/c/MnOQcM/uJyr), as in [4,5].

All scripts, fasta files and data files are available at https://github.com/ellasiera/Nat_res_vOTUs

*Soil properties*

Soil was weighed upon arrival to the lab (Wet Weight, WW). 10 g of soil were allowed to dry completely in a biological hood for up to several days depending on initial soil moisture, and weighed again (Dry Weight, DW). Soil water content was calculated as (WW-DW)/DW.

All other soil properties appearing in Table S1 were analyzed by Ward Laboratories (Kearney, NE, USA). Phosphorus and sulfate were extracted with Mehlich-3 buffer. Iron, manganese, copper, and zinc were extracted with DTPA buffer. Sodium, potassium, calcium, and magnesium were extracted with ammonium-acetate. Soil organic matter was calculated by percentage loss on ignition (LOI). Soil pH and soluble salts were measured using a 1:1 soil:water suspension. Nitrate was extracted with KCl.

*Data analysis*

Data analysis was performed in R on the vOTU read-mapped coverage table generated previously (see *Bioinformatics*). When calculating community similarity (beta-diversity), we used Jaccard similarity, as opposed to Bray-Curtis similarity, as there were so few vOTUs shared between samples. Similarity matrices were calculated using vegan [[17]](https://paperpile.com/c/MnOQcM/2O0S). Richness of vOTUs detected per site and per habitat was calculated using tidyverse [[18]](https://paperpile.com/c/MnOQcM/frO3) and plotted with cowplot [[19]](https://paperpile.com/c/MnOQcM/MpGq) and ggplot2 [[20]](https://paperpile.com/c/MnOQcM/Ud8O). Comparisons of vOTU richness between sites were performed with rstatix [[21]](https://paperpile.com/c/MnOQcM/uAaq), using the Games-Howell test to account for uneven sample size between habitats. Correlations between water content and viral richness, ANOVA comparison of water content between environments, and Tukey post hoc tests were calculated using the R stats package. The correlation analysis was performed under the hypothesis that higher moisture leads to higher richness and was therefore one-sided (alternative=greater). A Mantel test comparing viral community composition to soil chemistry was performed by creating Jaccard distance matrices (vegan function vegdist) and comparing them (mantel, 100 permutations; mantel.partial, 100 permutations) with the mantel and mantel.partial functions from the Vegan R package [[18]](https://paperpile.com/c/MnOQcM/2O0S).

*Data availability*

The datasets generated and/or analyzed in this study have been submitted to the NCBI sequence read archive (SRA) under BioProject number PRJNA831438 and will become available upon publication. The vOTU fasta sequences are available on https://github.com/ellasiera/Nat_res_vOTUs.

**Supplementary references**

1. [Santos-Medellín C, Estera-Molina K, Yuan M, Pett-Ridge J, Firestone MK, Emerson JB. Spatial turnover of soil viral populations and genotypes overlain by cohesive responses to moisture in grasslands. *bioRxiv* 2022. 2022.03.24.485562](http://paperpile.com/b/MnOQcM/Ioy5)

2. [Göller PC, Haro-Moreno JM, Rodriguez-Valera F, Loessner MJ, Gómez-Sanz E. Uncovering a hidden diversity: optimized protocols for the extraction of dsDNA bacteriophages from soil. *Microbiome* 2020; **8**](http://paperpile.com/b/MnOQcM/GrZe)**.1**: 1-16

3. [Emerson JB, Thomas BC, Andrade K, Allen EE, Heidelberg KB, Banfield JF. Dynamic viral populations in hypersaline systems as revealed by metagenomic assembly. *Appl Environ Microbiol* 2012; **78**: 6309–6320.](http://paperpile.com/b/MnOQcM/2mQot)

4. [Sorensen JW, Zinke LA, Ter Horst AM, Santos-Medellín C, Schroeder A, Emerson JB. DNase Treatment Improves Viral Enrichment in Agricultural Soil Viromes. *mSystems* 2021; **6**: e0061421.](http://paperpile.com/b/MnOQcM/WL2cq)

5. [Santos-Medellin C, Zinke LA, Ter Horst AM, Gelardi DL, Parikh SJ, Emerson JB. Viromes outperform total metagenomes in revealing the spatiotemporal patterns of agricultural soil viral communities. *ISME J* 2021; **15**: 1956–1970.](http://paperpile.com/b/MnOQcM/h9zg)

6. [Roux S, Emerson JB, Eloe-Fadrosh EA, Sullivan MB. Benchmarking viromics: an in silico evaluation of metagenome-enabled estimates of viral community composition and diversity. *PeerJ* 2017; **5**: e3817.](http://paperpile.com/b/MnOQcM/DFag2)

7. [Bolger AM, Lohse M, Usadel B. Trimmomatic: a flexible trimmer for Illumina sequence data. *Bioinformatics* 2014; **30**: 2114–2120.](http://paperpile.com/b/MnOQcM/lPE9N)

8. [Bushnell B. BBTools software package. *URL http://sourceforge net/projects/bbmap* 2014.](http://paperpile.com/b/MnOQcM/vjLTq)

9. [Ter Horst AM, Santos-Medellín C, Sorensen JW, Zinke LA, Wilson RM, Johnston ER, et al. Minnesota peat viromes reveal terrestrial and aquatic niche partitioning for local and global viral populations. *Microbiome* 2021; **9**: 233.](http://paperpile.com/b/MnOQcM/kP2n)

10. [Li D, Liu C-M, Luo R, Sadakane K, Lam T-W. MEGAHIT: an ultra-fast single-node solution for large and complex metagenomics assembly via succinct de Bruijn graph. *Bioinformatics* 2015; **31**: 1674–1676.](http://paperpile.com/b/MnOQcM/10aEf)

11. [Kieft K, Zhou Z, Anantharaman K. VIBRANT: automated recovery, annotation and curation of microbial viruses, and evaluation of viral community function from genomic sequences. *Microbiome* 2020; **8**: 90.](http://paperpile.com/b/MnOQcM/opYBg)

12. [Olm MR, Brown CT, Brooks B, Banfield JF. dRep: a tool for fast and accurate genomic comparisons that enables improved genome recovery from metagenomes through de-replication. *ISME J* 2017; **11**: 2864–2868.](http://paperpile.com/b/MnOQcM/CTBYt)

13. [Langmead B, Salzberg SL. Langmead. 2013. Bowtie2. *Nat Methods* 2013; **9**: 357–359.](http://paperpile.com/b/MnOQcM/giCft)

14. [BamM: Metagenomics-focused BAM file manipulation. Github.](http://paperpile.com/b/MnOQcM/dNTQD)

15. [Kopylova E, Noé L, Touzet H. SortMeRNA: fast and accurate filtering of ribosomal RNAs in metatranscriptomic data. *Bioinformatics* 2012; **28**: 3211–3217.](http://paperpile.com/b/MnOQcM/s2Ub)

16. [Quast C, Pruesse E, Yilmaz P, Gerken J, Schweer T, Yarza P, et al. The SILVA ribosomal RNA gene database project: improved data processing and web-based tools. *Nucleic Acids Res* 2013; **41**: D590–6.](http://paperpile.com/b/MnOQcM/uJyr)

17. [Oksanen J, Blanchet FG, Kindt R, Legendre P, Minchin PR, O’hara RB, et al. Vegan: community ecology package. R package version 2.3-0; 2015. *Sci Rep* 2020; **10**: 20354.](http://paperpile.com/b/MnOQcM/2O0S)

18. [Wickham H, Averick M, Bryan J, Chang W, McGowan L, François R, et al. Welcome to the tidyverse. *J Open Source Softw* 2019; **4**: 1686.](http://paperpile.com/b/MnOQcM/frO3)

19. [Wilke CO. cowplot: streamlined plot theme and plot annotations for ‘ggplot2’. *R package version 0 9* 2019; **4**.](http://paperpile.com/b/MnOQcM/MpGq)

20. [Wickham H. ggplot2: Elegant Graphics for Data Analysis. 2016. Springer.](http://paperpile.com/b/MnOQcM/Ud8O)

21. [Kassambara A. rstatix: Pipe-friendly framework for basic statistical tests. *R package version 0 6 0* 2020.](http://paperpile.com/b/MnOQcM/uAaq)
