# Supplementary material for: Substantial differences in soil viral community composition within and among four Northern California habitats
Source: ISME Commun. 2022 Oct 13;2:100. doi: 10.1038/s43705-022-00171-y (PMC9723544; doi:10.1038/s43705-022-00171-y)
Supplement: Supplementary file 3 — Supplementary Table S2 [file 43705_2022_171_MOESM3_ESM.docx]

Table S2. Summary of assembly statistics including detection of viral genomes per habitat

|  | Across all habitats | Grassland | Wetland | Woodland | Chaparral |
| --- | --- | --- | --- | --- | --- |
| Total reads | 2,160,621,059 | 943,863,746 | 577,454,144 | 358,356,831 | 280,946,338 |
| Total contigs > 10 kbp | 12,850 | 3,376 | 8,513 | 704 | 257 |
| Average reads per virome | 72,950,833 | 67,401,933 | 82,493,449 | 71,671,366 | 70,236,585 |
| Average contigs > 10 kbp per virome | 416 | 241 | 1,216 | 141 | 64 |
| Average viral contigs predicted per virome (VIBRANT) | 116 | 68 | 307 | 39 | 50 |
| Total viral contigs predicted (VIBRANT) | 3,490 | 949 | 2,148 | 195 | 198 |
