# Supplementary material for: Substantial differences in soil viral community composition within and among four Northern California habitats
Source: ISME Commun. 2022 Oct 13;2:100. doi: 10.1038/s43705-022-00171-y (PMC9723544; doi:10.1038/s43705-022-00171-y)
Supplement: Supplementary file 4 — Supplementary Table S3 [file 43705_2022_171_MOESM4_ESM.docx]

Table S3. Distance (km) between UC Davis Natural Reserves sites sampled

|  | QR | SCC | JP | ML | BB |
| --- | --- | --- | --- | --- | --- |
| QR |  | 6 | 36 | 50 | 85 |
| SCC |  |  | 36 | 50 | 85 |
| JP |  |  |  | 85 | 109 |
| ML |  |  |  |  | 85 |
