# Supplementary material for: Substantial differences in soil viral community composition within and among four Northern California habitats
Source: ISME Commun. 2022 Oct 13;2:100. doi: 10.1038/s43705-022-00171-y (PMC9723544; doi:10.1038/s43705-022-00171-y)

Supplementary figure 1: Map of sampling sites in Northern California colored by habitat

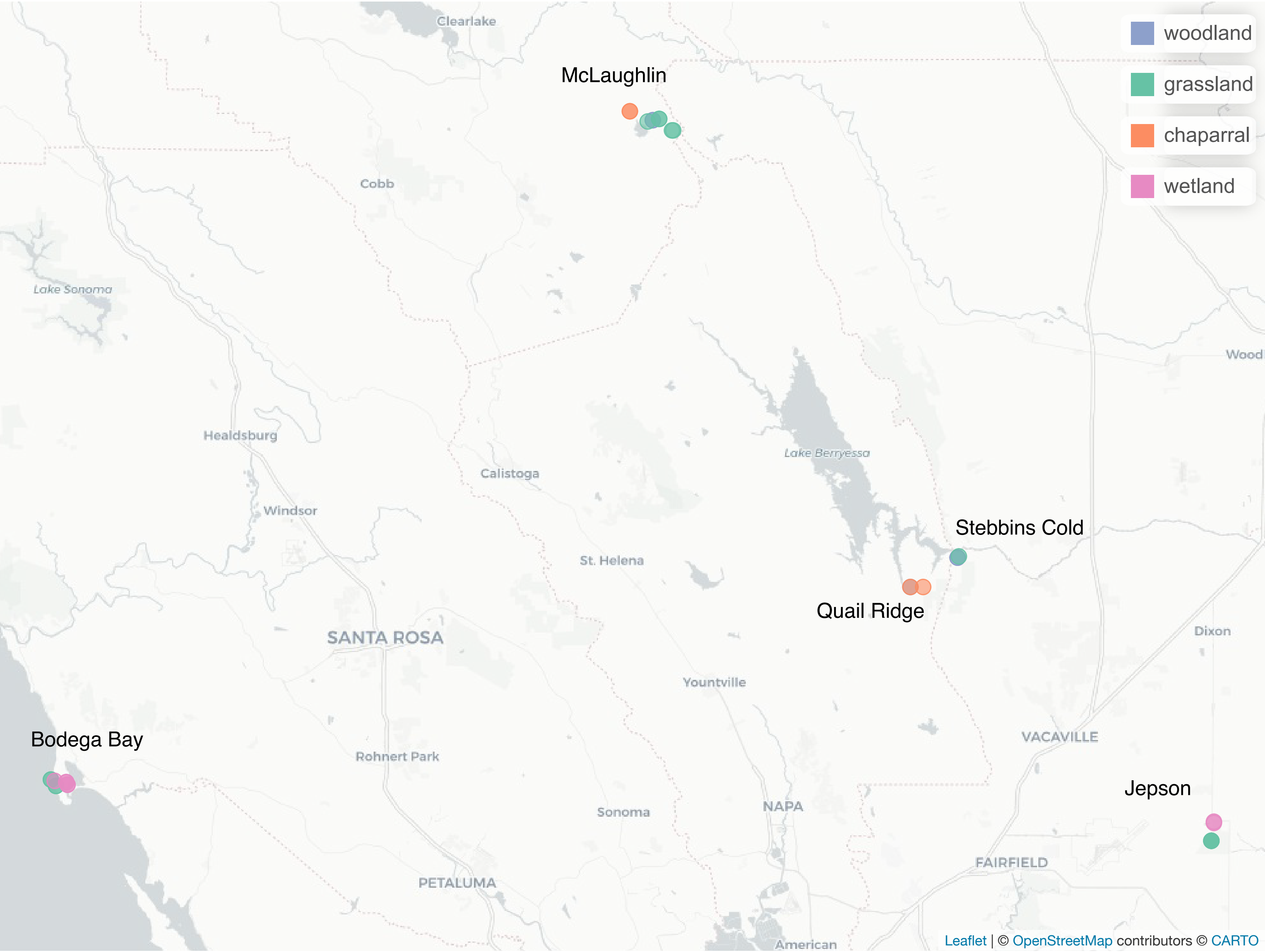

Supplementary figure 2: Beta diversity of viral communities

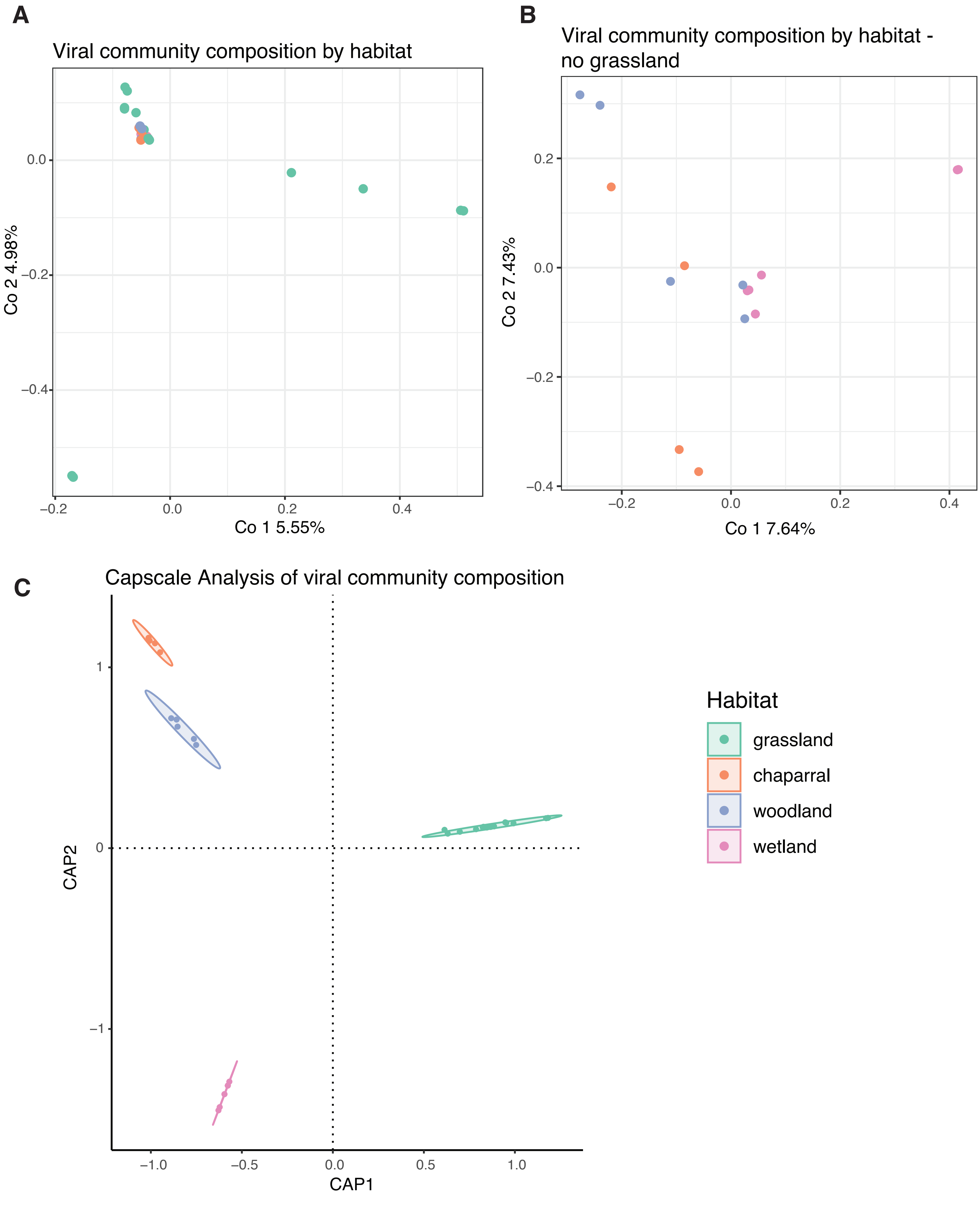

Supplementary figure 3: vOTUs unique to or shared between viromes and habitats

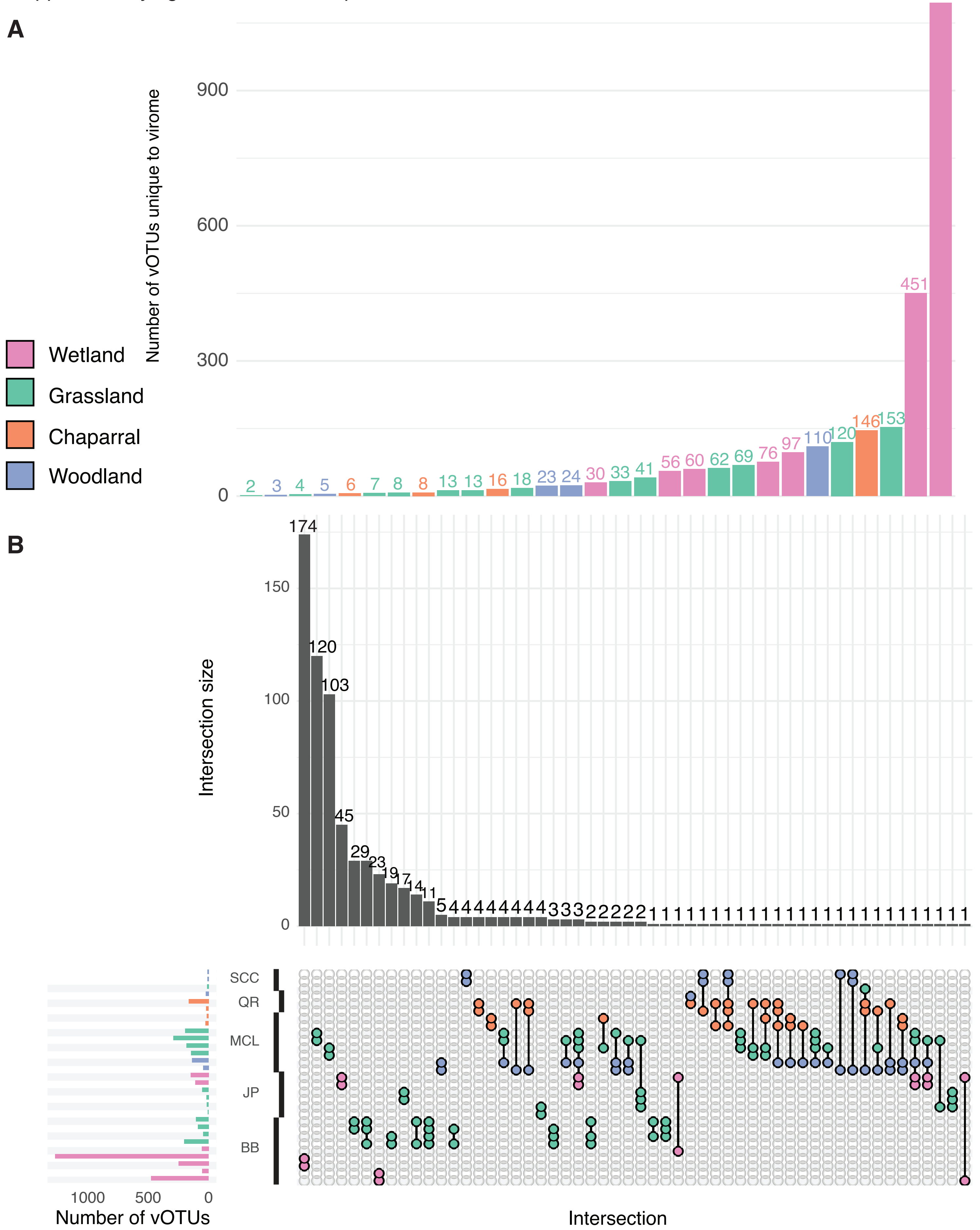

Supplement: Supplementary file 6 — Supplementary Figures [file 43705_2022_171_MOESM6_ESM.pdf]
